# Evaluation of the relationship between malar projection and lower facial convexity in terms of perceived attractiveness in 3-dimensional reconstructed images

**DOI:** 10.1186/s13005-020-00223-5

**Published:** 2020-04-28

**Authors:** Hon Kwan Woo, Deepal Haresh Ajmera, Pradeep Singh, Kar Yan Li, Michael Marc Bornstein, Kwan Lok Tse, Yanqi Yang, Min Gu

**Affiliations:** 1 Faculty of Dentistry, The University of Hong Kong, Hong Kong SAR, China; 2 Central Research Laboratories, Faculty of Dentistry, The University of Hong Kong, Hong Kong SAR, China; 3 Applied Oral Sciences and Community Dental Care, Faculty of Dentistry, The University of Hong Kong, Hong Kong SAR, China; 4 Department of Oral Health & Medicine, University Center for Dental Medicine Basel UZB, University of Basel, Basel, Switzerland

**Keywords:** Malar, Lower facial convexity, Jaws, 3-dimensional, CBCT

## Abstract

**Background:**

This study aimed to investigate dental student’s perception of facial attractiveness with regard to different combinations of anteroposterior malar-jaw positions using 3-dimensional (3-D) reconstructed images of subjects.

**Methods:**

Two Chinese young adults (1 male and 1 female) with straight profiles and average malar projections were selected for the study. 3-D facial images and cone-beam computed tomography images of these two subjects were superimposed using 3-D imaging software. Lateral and oblique views of nine different images were created by moving the maxillomandibular complex and/or zygomatic bone by 4 mm either forward or backward along the sagittal plane. One hundred three undergraduate dental students (*n* = 24, 33, and 46 students from the Year 3, 4, and 5, respectively) then scored lateral and 45° oblique view images of the newly reconstructed faces.

**Results:**

In the present study, images with a neutral malar and retruded jaws were found to be the most attractive in both male and female subjects. In addition, the Protruded malar (PM) group (*p* < 0.001), and the Retruded Jaws (RJ) group were rated more attractive (*p* < 0.001). Furthermore, the Relatively Prominent malar (RP) group was rated more attractive (*p* < 0.001) when malar-jaw relative positions were compared.

**Conclusion:**

This study shows that a neutral or a protruded malar favours facial attractiveness in both Chinese male and female subjects. Therefore, an appropriate relationship between malar projection and lower facial convexity should be taken into consideration while designing the orthodontic/orthognathic treatment plans for enhanced aesthetic outcomes.

## Introduction

Beauty and harmony are among those quantifiable objective facial characteristics that humans seek and long for [1] as they play an inherent role in social behaviour and perception worldwide [2]. The increased awareness of facial aesthetics has also led to an increase in the number of patients seeking orthodontic/orthognathic treatment [3, 4], thus providing the orthodontists and maxillofacial surgeons with an opportunity to significantly enhance a person’s appearance. Therefore, a deep understanding of these desired characteristics is required.

Three distinct promontories including the nose, malar eminences, and chin determine a person’s middle and lower third facial characteristics [5]. Adequate balance among these facial promontories is what is required to achieve facial harmony [6]. Malar contour plays a crucial role in defining the shape of the lateral segment of the middle third of the face [7]. Being the widest point on the face, malar eminence is an important factor while determining facial attractiveness [8]. Rounded and thick malar contours are considered to be attractive among Caucasians [7, 9] whereas, a slender and ovoid face is perceived to be youthful and pleasing by Asians [10]. Accentuation of malar area enhances the angularity and provides fullness to the midface [11], on the other hand, underdevelopment of this region may lead to hypoplasia of middle third of the face, imparting a certain degree of flatness to the face and thereby contributing to an elderly look [7, 9, 12, 13].

Amongst the various components that make up the facial skeleton, the zygoma and maxillomandibular complex are considered to be the key components, determining the perceived shape of the face. An anteriorly projected malar–midfacial complex has to be accomplished for the enhancement of facial aesthetics [14, 15]. On the contrary, a prominent mandibular angle when combined with a protruding zygoma, characteristically produces a quadrangular, obstinate and masculine appearance [16]. Hence, a balance between the relative malar-jaw positions is what is required to achieve facial harmony. A multitude of surgical techniques exist for malar reduction [17] and augmentation [13] in addition to fillers [18, 19] and implants. Where LeFort osteotomies have been used as a viable treatment option for the correction of functional and aesthetic manifestations of malar deficiency associated with maxillary protrusion [20], several authors have also attempted different modifications in the osteotomy approaches for malar reduction and mandibular reshaping to achieve an ideal facial shape [21]. Consequently, the mandibular angle and/or reduction malarplasty have become the most frequently performed procedures for aesthetic facial-bone contouring in Asian countries [21].

A comprehensive analysis of facial traits and supporting structures is central to enhance diagnosis, treatment planning, and quality of results [22]. Given the fact that both malar [23, 24] and the jaws [25] are among defining components of the facial profile, any discrepancy in the balance between dentoalveolar and malar support may result in distorted nasal base-lip contour (Nb-LC), which may further compromise youthful appearance [24, 26]. Several previous studies have investigated the effect of different jaw profiles (bimaxillary protrusion, retrusion, straight profiles, retrognathism, prognathism) regarding facial aesthetics [27, 28]. Generally, these studies identified a straight jaw profile and bimaxillary retrusion as the most attractive among both Caucasian [28] and Asian-Chinese subjects [27]. However, increasing attention has recently been directed towards the aesthetic impact of malar projection/reduction on facial appearance [29, 30].

Since the relative positions of malar and jaw have never been investigated, this study aimed 1) to investigate how different combinations of anteroposterior malar-jaw positions affect the subjective facial aesthetics as assessed by dental students; 2) to investigate whether there are any significant differences in the perception of attractiveness by male and female dental students. We hypothesize that the faces with an equal or positive relationship between malar projection and jaw protrusion would be more attractive than the faces with a negative relationship.

## Materials and methods

### Subject selection and image acquisition

In the present study, two Chinese subjects (1 male and 1 female, aged 20 years) with (a) straight profile; (b) average malar projection of approximately 2 mm beyond the anterior surface of the cornea [24, 31]; (c) with no previous history of orthognathic surgery and (d) no facial anomalies, were selected from the orthodontic patient pool of the Faculty of Dentistry, University of Hong Kong. Cone-beam computed tomography (CBCT) and three-dimensional (3-D) images of both the subjects were obtained within a month after the completion of orthodontic treatment.

3-D facial images of both the subjects were captured with Morpheus 3D scanner (Morpheus Co., Ltd., Korea). The patients were scanned for approximately 0.8 s while sitting upright with the head in a natural position and lips closed. Also, each subject underwent a CBCT scan using Planmeca (Planmeca, ProMax 3D Mid, Planmeca Oy Inc., Finland) with the full field of view (20.0 × 17.4 cm), 0.4 mm voxels, and with two 4.8 s scans to capture the complete dataset.

### Image processing

**Fig. 1:**
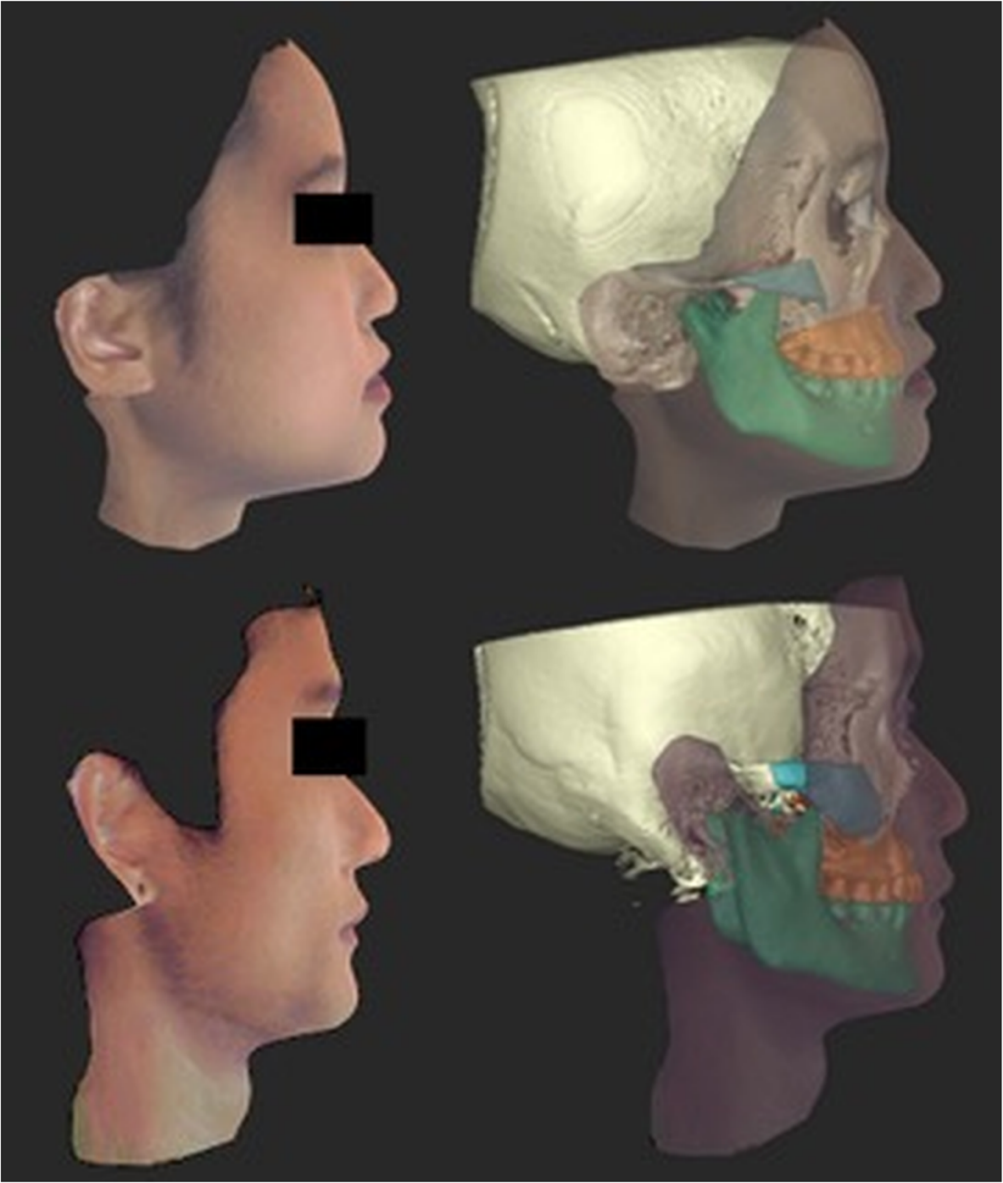
Superimposition of facial 3D images on CBCT images

The CBCT data was imported to Morpheus 3D Dental Solution software (Morpheus Co., Ltd., Korea) as DICOM (Digital Imaging and Communications in Medicine) files, followed by the superimposition of the 3D facial image on the soft tissue surface of CBCT using automatic and manual adjustment functions of the software (Fig. 1). Subsequently, osteotomy cuts on zygoma, maxilla, and mandible, were planned so as to simulate the osseous movements along the sagittal plane i.e. advancement (+ 4 mm) and/or set-back (− 4 mm) with respect to zygoma and maxillomandibular complexes (greater than 1 standard deviation of the norms) [32]. After the simulated movement, the 3D facial images morphed accordingly. The ratio of soft to hard tissue changes used the system’s default setting, which was in accordance with previous literature [33, 34].

### Assessors

With reference to a previous study [27], a sample size of 98 was calculated using G*Power (version 3.1.9.2, Kiel University, Germany) with a power of 0.9 and alpha of 0.05 to detect the perception of attractiveness by dental students. For the present study, a total of 103 Chinese dental students (45 males and 58 females, with a mean age of 22.5 ± 1.5 years) from the year 3 (*n* = 24), year 4 (*n* = 33) and year 5 (*n* = 46) were recruited from the faculty of dentistry, University of Hong Kong.

### Attractiveness rating

**Fig. 2:**
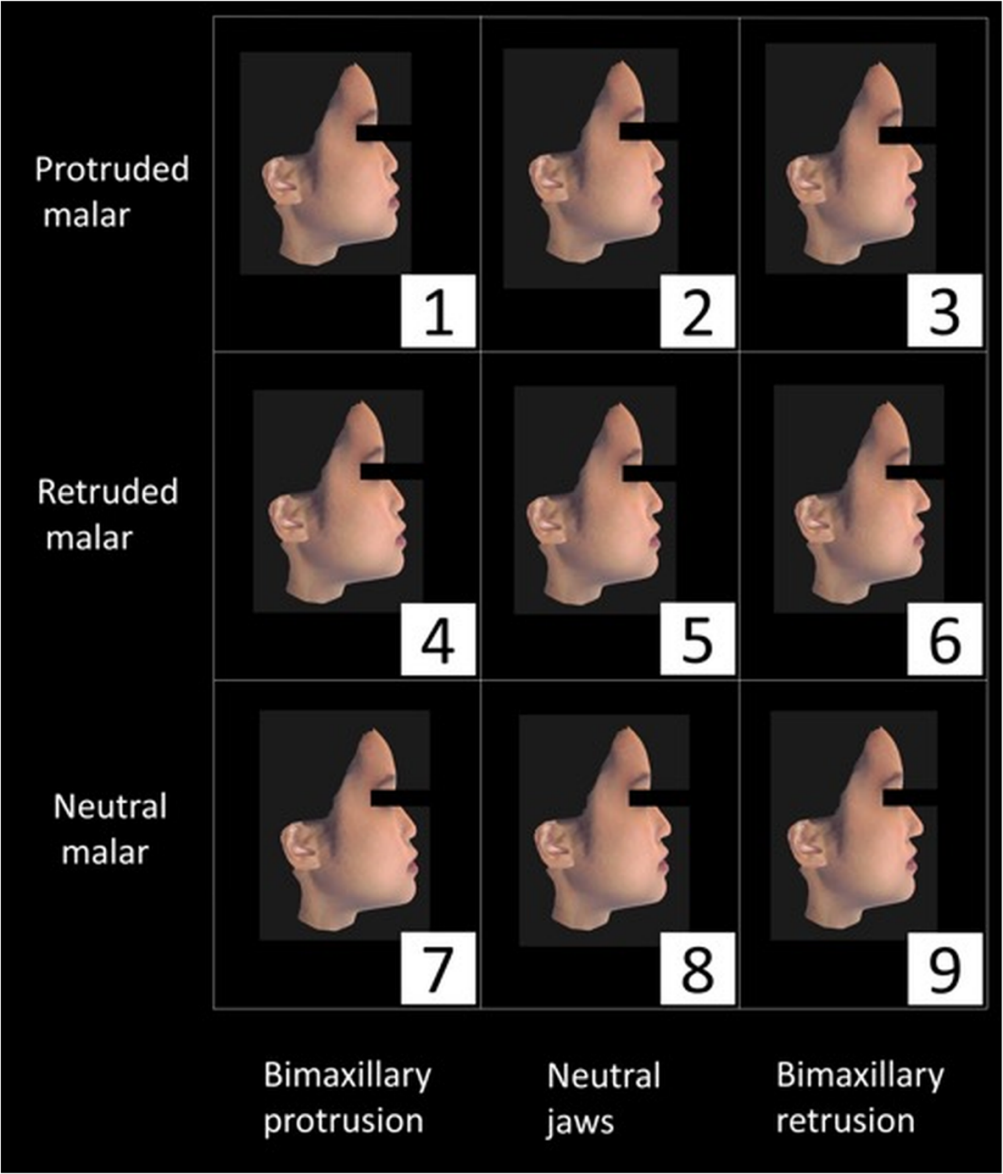
Lateral view of 9 reconstructed female faces

**Fig. 3:**
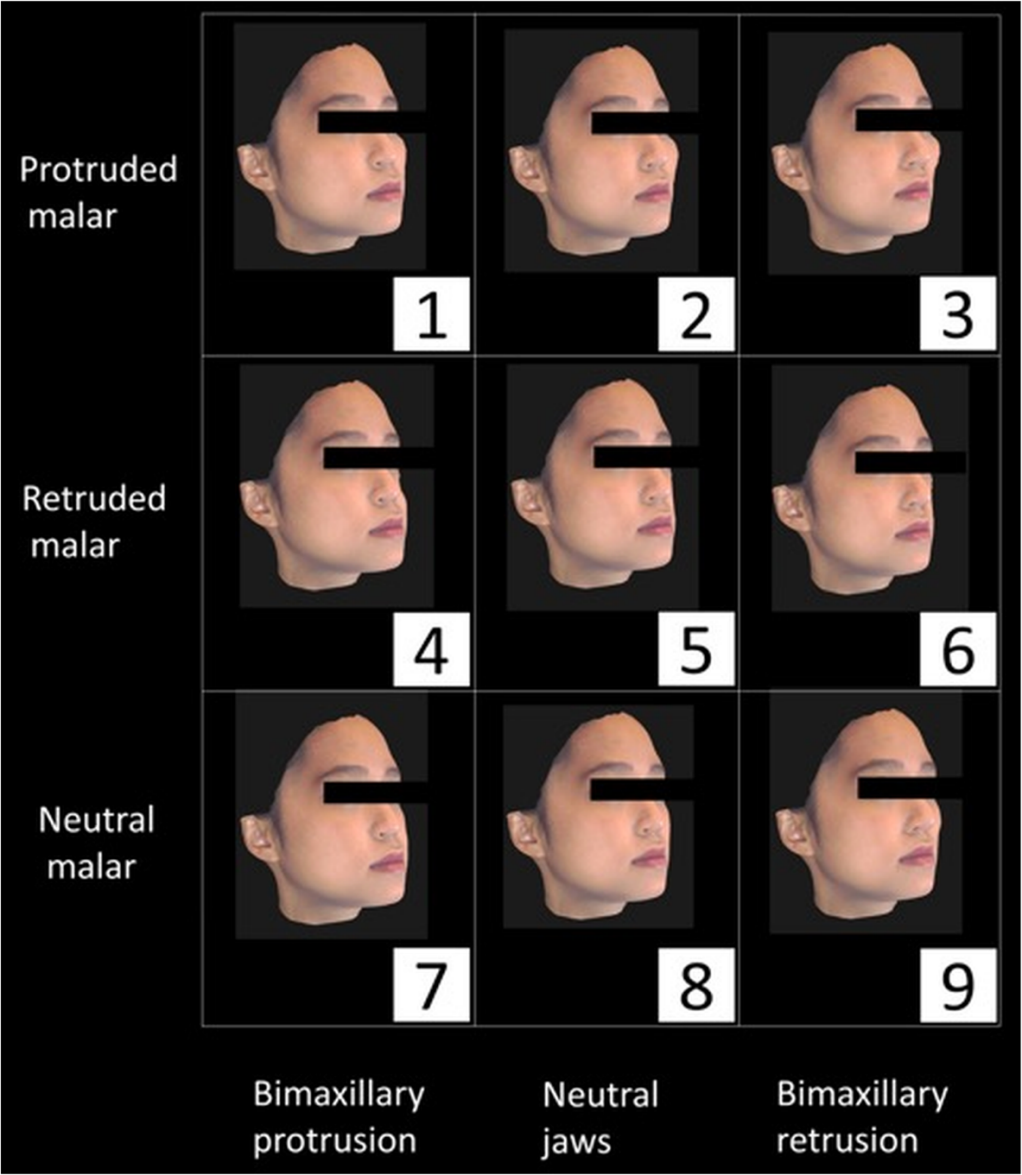
Oblique view of 9 reconstructed female faces

**Fig. 4:**
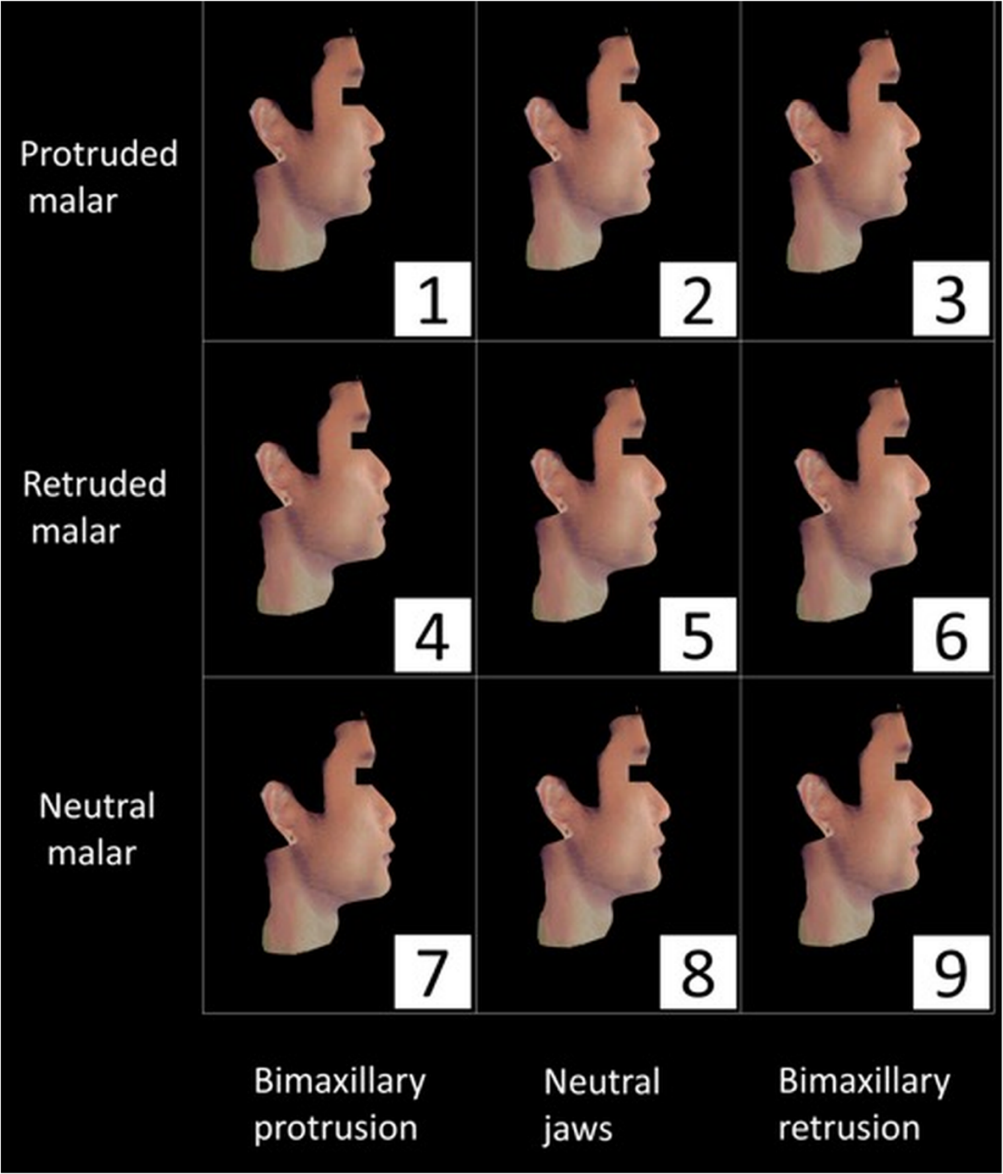
Lateral view of 9 reconstructed male faces

**Fig. 5:**
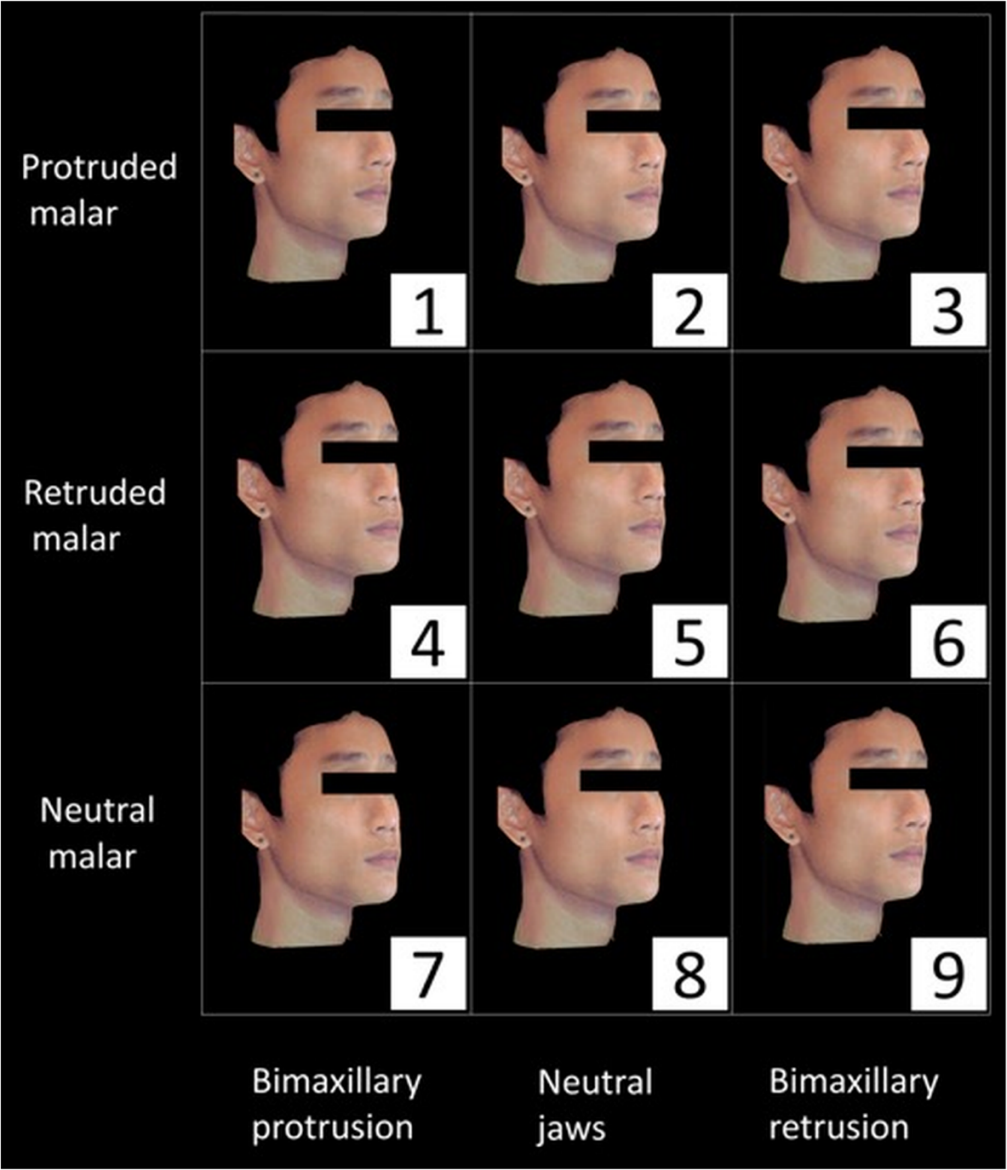
Oblique view of 9 reconstructed male faces

The assessors were presented with reconstructed faces in the lateral and 45° oblique views (Figs. 2, 3, 4 and 5). To ease the process of comparing the 9 images simultaneously, the reconstructed images were arranged in a systematic order and the observers were asked to rank the images in the horizontal and vertical directions. Jaw protrusion varied along the rows (i.e. horizontally), while malar projection varied along the columns (i.e. vertically). The images in each row were assigned a non-repetitive horizontal score (X score; 1–3: 1 = most attractive, 3 = least attractive). Likewise, images in each column were assigned a vertical score (Y score).

The questionnaire was designed accordingly, to compare the images only in the horizontal and vertical directions. In addition, observers were also questioned as to which part of the face (forehead, nose, upper and lower lips, malar) strongly influenced their decision.

### Data analysis

Each image received a horizontal and vertical score (X score, Y score) and for every image, a combined score was calculated as X score × Y score. Furthermore, an overall score was computed as (2 × combined score oblique + 1 × combined score lateral) for all faces. The oblique combined score was double-weighted, as usually, faces are viewed at an angle during social interactions [35].

To investigate the relative malar deficiency and prominence, the authors introduced a new concept – The contrast between malar and jaw deviations (from the average) i.e. ***“Malar-Jaw-Contrast”*** which was calculated as MJC = Malar deviation* – Jaw deviation* (mm).

*deviation was measured in relation to the original faces which had average malar projection and jaw positions (Neutral, N). In the present study, the deviation was + 4 mm. Table 1, represents features of reconstructed faces after simulated advancement and/or set-back movements.

**Table 1:** Features of the 9 reconstructed faces after simulated advancement and/or set-back movements

Face No.	Malar position deviation from original image* (mm)	Jaw position deviation from original image^a^ (mm)
1	+ 4	+ 4
2	+ 4	0
3	+ 4	−4
4	−4	+ 4
5	−4	0
6	−4	− 4
7	0	+ 4
8	0	0
9	0	−4

^a^positive = advancement; negative = setback; 0 = no change

For the aforementioned evaluation, 9 reconstructed faces were classified into three groups viz.; Relatively Deficient malar (RD; the malar was retruded from neutral or the jaws were protruded), Relatively Prominent malar (RP; the malar was protruded from neutral or the jaws were retruded) and Balanced Profile (BP; the malar and jaws were protruded/retruded in the same direction) (Table 2).

**Table 2:** Classification of faces according to simulated movements

Group	Facial images (no.)	Value of MJC (mm)
Relatively prominent malar (RP)	2	+ 4
	3	+ 8
	9	+ 4
Balanced profile (BP)	1	0
	6	0
	8	0
Relatively deficient malar (RD)	4	−8
	5	−4
	7	−4

The present study was conducted in full accordance with the Declaration of Helsinki 2013 (www.wma.net) after obtaining study protocol approval from the local institutional review board (IRB) of the University of Hong Kong.

### Statistics

To ensure the reproducibility, thirty randomly selected students were asked to complete the questionnaire yet again after an interval of at least 2 weeks. A one-way intra-class correlation coefficient was used to determine the intra-observer reliability that showed a satisfactory agreement among the overall scores (0.658).

The Shapiro-Wilk test was used to evaluate the normality of the data and non-parametric tests were adopted for non-normally distributed data. To judge the overall scores given by males against the scores allotted by females, a Mann–Whitney test was performed. Further, differences in the overall scores were evaluated using the Kruskal–Wallis test and post hoc pairwise comparisons. Also, Fisher’s exact test was used to analyse the distribution of the most influential facial parts in terms of rating.

The Bonferroni correlation was used for multiple comparisons in the pairwise and subgroup analysis. After considering the number of outliers (i.e. the mean was likely to misrepresent the average), the median was used rather than mean. Therefore, the data in this study are presented as medians and standard deviations. All statistical tests were performed using SPSS software (IBM SPSS Statistics 20, IBM Corp., USA) with a statistical significance level α = 0.05.

## Results

### Difference between female and male observers

The Mann–Whitney test showed no significant difference between male and female observers (*p* > 0.05).

### Differences among the 9 facial images

**Fig. 6:**
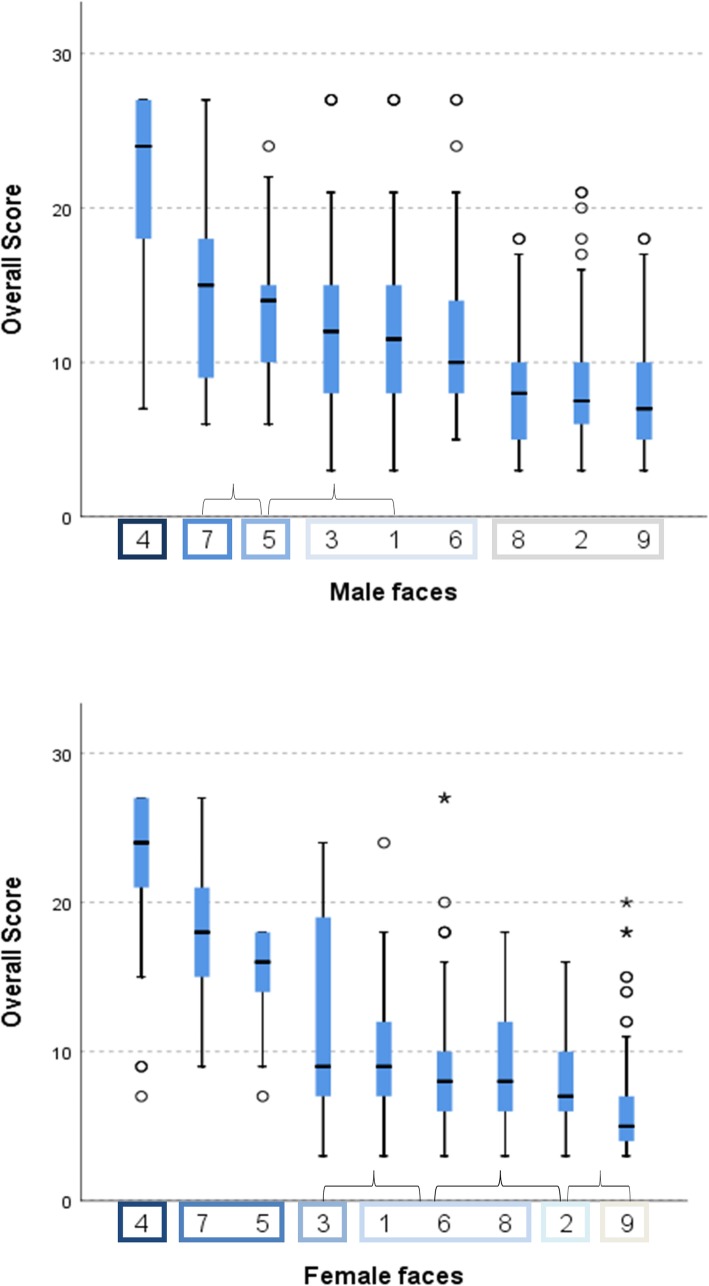
Box plot representation of Kruskal–Wallis analysis of the overall scores of the 9 reconstructed faces. The interior bars indicate the medians while the error bars display the inner limits (1.5 X interquartile range). The rectangular boxes denote the homogeneity subset. Identical letters imply non-significant differences, while different letters infers significant differences (Kruskal–Wallis test*, P < 0.05)*

The results of a Kruskal–Wallis analysis of the overall facial scores are shown in Fig. 6. Among the images of the male subject, No. 9 (neutral malar, retruded jaws) received a lower overall score (more attractive) than all other faces (all *p* < 0.001) except No. 2 (protruded malar, neutral jaws) and No. 8 (neutral malar, neutral jaws). In contrast, image No. 4 (retruded malar, protruded jaws) received the highest overall score (i.e. most unattractive) (all *p* < 0.001), followed by image No. 7 (neutral malar, protruded jaws). Correspondingly, among the images of the female subject, image No. 9 was rated a significantly lower overall score compared to all other images (all *p* < 0.01) except No. 2. Further, No. 4 received the highest overall score (all *p* < 0.01), followed by image No. 5 (retruded malar, neutral jaws) and No. 7.

### Comparison of malar position

For both the male and female subjects, images with retruded malar (RM) received a significantly higher score (i.e. most unattractive) as compared to other positions of malar (Kruskal–Wallis test, all *p* < 0.001). However, no significant difference was observed between the protruded (PM) and neutral malar groups (NM) (all *p* > 0.05) (Table 3).

**Table 3:** Inter-group comparisons

	Overall score (Median (Standard deviation))			*p* value	Bonferroni post hoc *p* value		
Malar position group	Protruded Malar (PM)	Retruded Malar (RM)	Neutral Malar (NM)		PM vs. RM	PM vs. NM	RM vs. NM
M	9.0 (5.3)	14.0 (6.6)	9.0 (5.5)	*p* < 0.001***	*p* < 0.001***	*p* = 0.545	*p* < 0.001***
F	9.0 (4.6)	16.0 (7.2)	10.0 (5.9)	*p* < 0.001***	*p* < 0.001***	*p* = 0.542	*p* < 0.001***
M vs. F	*p* = 0.735	*p* = 0.003*	*p* = 0.045*				
Jaw position group	Protruded Jaws (PJ)	Retruded Jaws (RJ)	Neutral Jaws (NJ)		PJ vs. RJ	PJ vs. NJ	RJ vs. NJ
M	15.0 (6.7)	9.0 (5.2)	9.0 (4.5)	*p* < 0.001***	*p* < 0.001***	*p* < 0.001***	*p* = 1.000
F	18.0 (7.0)	7.0 (5.1)	10.0 (4.4)	*p* < 0.001***	*p* < 0.001***	*p* < 0.001***	*p* < 0.001***
M vs. F	*p* = 0.159	*p* = 0.987	*p* = 0.801				
Relative position group	Relatively deficient malar (RD)	Balanced profiles (BP)	Relatively prominent malar (RP)		RD vs. BP	BP vs. RP	RP vs. RD
M	15.0 (6.2)	9.0 (5.0)	8.0 (4.9)	*p* < 0.001***	*p* < 0.001***	*p* = 0.033*	*p* < 0.001***
F	18.0 (5.4)	9.0 (3.8)	7.0 (4.8)	*p* < 0.001***	*p* < 0.001***	*p* = 0.063	*p* < 0.001***
M vs. F	*p* < 0.001***	*p* = 0.096	*p* = 0.162				

*M* Male subject, *F* Female subject; all comparisons were Bonferroni-adjusted; * *p* < 0.05, *** *p* < 0.001

### Comparison of lower facial convexity (jaws)

In both the male and female subjects, the observers assigned a significantly higher score (i.e. most unattractive) to the protruded jaw group (PJ) as compared to other groups (Kruskal–Wallis test, both *p* < 0.001). Furthermore, a significant difference was observed between the retruded jaw (RJ) and neutral jaw group (NJ) of the female subject (*p* < 0.001), but not the male subject (*p* > 0.05) (Table 3).

### Comparison of the RP, RD and BP groups

While evaluating the contrast positions between the malar and jaw, image No. 4 (retruded malar, protruded jaws; MJC_No.4_ = − 4 – (+ 4) = − 8 mm) showed more negative Malar-Jaw-Contrast, i.e. relatively more malar-deficient compared to No. 5 (retruded malar, neutral jaws; MJC_No.5_ = − 4 -0 = − 4 mm) and No. 7 (neutral malar, protruded jaws; MJC_No.7_ = 0 – (+ 4) = − 4 mm), whereas the latter 2 images shared the same degree of relative malar deficiency. In particular, the RP faces No. 9 (neutral malar, retruded jaws; MJC = + 4) and No. 2 (protruded malar, neutral jaws; MJC = + 4) had less prominent malars relative to No. 3 (protruded malar, retruded jaws; MJC = + 8)) but were rated as relatively more attractive. For both the female and male subjects, the following overall score sequence was identified: RP < BP < RD. For the male subject, significant differences were observed between all the groups (all *P* < 0.05). However, no significant difference was observed between BP and RP for the female subject (Table 3).

### Comparison of images of male and female subjects within groups

The overall scores received by the male and female subjects differed significantly in the PM (*p* < 0.01) and NM groups (*p* < 0.05), however, no significant differences were observed within the groups of altered jaw position (Table 3). Notably, the male subject received a lower overall score than the female subject in the RD group, whereas the scores did not differ by subject gender in the BP and RP groups (Table 3).

### Most influential part

Table 4, presents the distribution of the “most influential part” as identified by the observers. The malar and lips were considered to be extremely influential features according to 47.2 and 43.5% of the observers, respectively. However, no significant difference was observed between the male and female subjects (Fisher’s exact test, *p* > 0.05) concerning the distribution of the most influential features.

**Table 4:** Features considered most influential while rating images of the female and male subjects

			Male	Female	Total	*p* value
Features	Forehead	Count	4	3	7	
		Percentage	2.1%	1.5%	1.8%	
	Nose	Count	20	9	29	
		Percentage	10.4%	4.6%	7.5%	
	Lips	Count	82	86	168	
		Percentage	42.7%	44.3%	43.5%	
	Malar	Count	86	96	182	
		Percentage	44.8%	49.5%	47.2%	
Total		Count	192	194	386	
		Percentage	100.0%	100.0%	100.0%	0.163

## Discussion

Pleasant aesthetics is characterized by the harmony and correct balance between the promontories that make up the facial profile, therefore, proper identification of unpleasant facial features, a thorough understanding of the patient’s priorities and a comprehensive preoperative assessment of the facial traits is central to ensure greater patient satisfaction post orthodontic/surgical procedure. High-quality 3-D images play an imperative role in the diagnostic evaluation, however, a previous study by Zhu et al. reported that 3-D images could not be viewed in true 3-D on screens unless they are projected stereotypically [36], therefore, 3-D rotatable images were used for the current study. For this purpose, CBCT images were merged with 3-D facial images prior to the assessment. Advantageously, this process allowed a simulation of 3-D changes that occur in faces subjected to orthognathic and malar surgeries [29, 37].

**Fig. 7:**
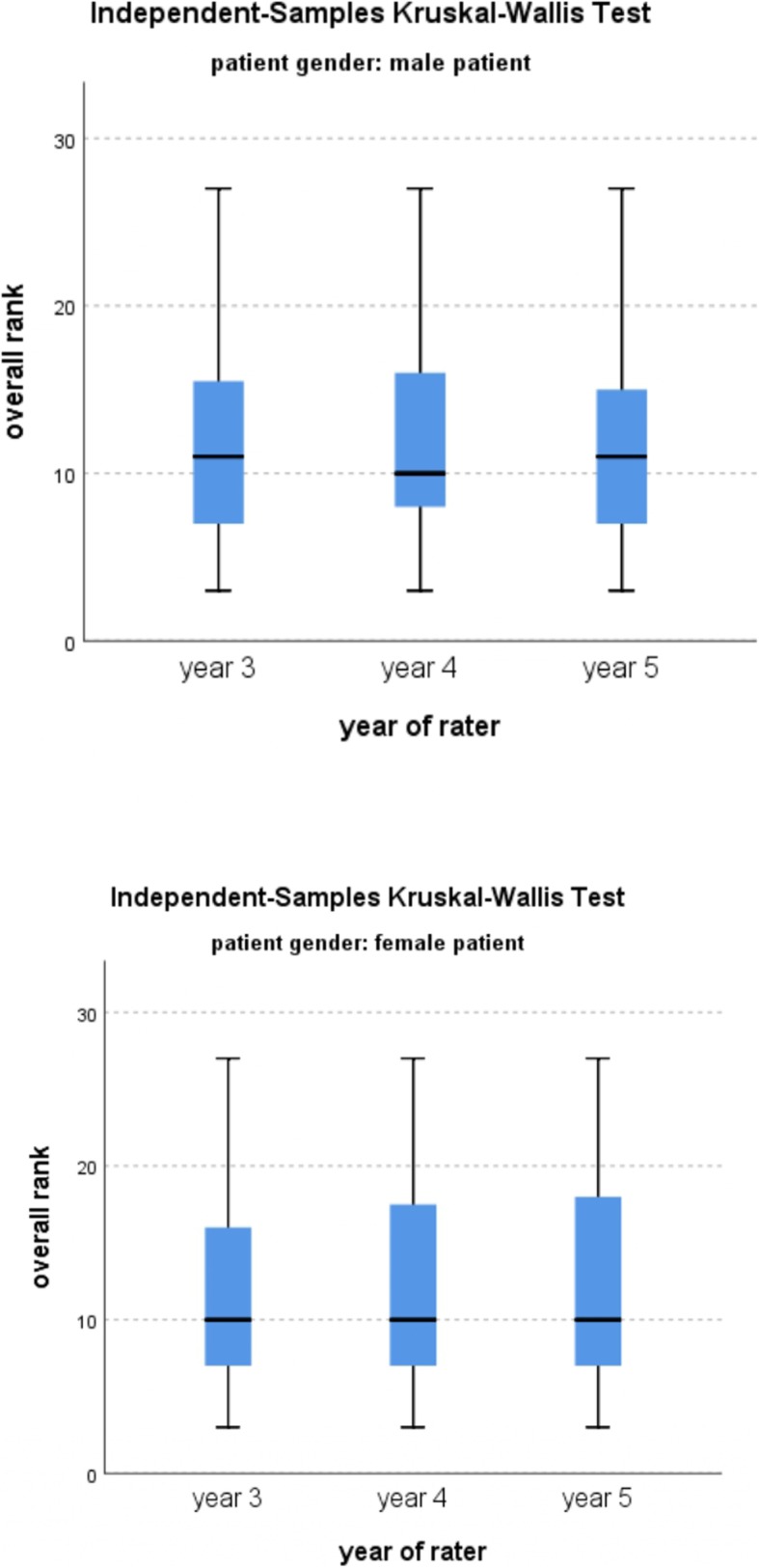
Box plot representation of Independent-samples Kruskal–Wallis analysis of the overall rank vs the categories of the year of rater *(Kruskal–Wallis test, P < 0.05)*

Assessment of facial aesthetics often involves facial scoring surveys involving professionals and/or laymen. As a consequence of training and influences from western norms, dental professionals often place a higher emphasis on a straight profile as compared to laymen [27, 28]. On the other hand, laymen also tend to prefer a straight profile, nevertheless the preference is rather less pervasive [27, 28]. Correspondingly for the present survey, dental students were regarded as ideal image observers because their perception of facial aesthetics would be expected to fall between those of professionals and laymen and their judgments would, therefore, comprise both academic and intuitive components. Ideally, the detailed knowledge regarding face assessment is learned during the specialist training phase of orthodontics or orthognathic surgery across various universities around the world. In our university, the undergraduate program is of 6 years, and the curriculum has been designed to introduce the orthodontics and orthognathic surgery from the year 4. However, the didactic component for orthodontics and orthognathic surgery is very limited and there is no difference between the orthodontics and orthognathic surgery knowledge of year 3, 4 and 5 students. In order to elucidate this fact, when the ratings between the year 3, 4 and 5 students were compared, no differences were noticed (Fig. 7). Therefore, the aesthetic perception of the students from year 3, 4 and 5 can be considered intermediate to professionals and the lay persons, but more comparable to lay persons, which was in agreement to the findings of the previous studies [27]. Hence the present study evaluates the perception of dental students about implicit facial aesthetic traits and perceived malar-jaw positions.

Till now researchers have mainly focused on lateral facial profile for analysing facial aesthetics [23, 27, 28, 38]. For the current study, the observers were asked to score the faces in both the oblique and lateral views to provide a more complete facial comparison. In our visual cortex, the oblique view, which is presented in most social interactions and has therefore been referred as the “social profile” [7], maybe more important than the lateral view while assessing the relationship between malar [39] and the jaws, since it not only provides facial information, such as malar, nose and chin protrusion but also makes a portion of the frontal view visible. Besides, this three- quarter profile provides a more natural, comprehensive and better impression of the facial profile to be evaluated [40–42]. Therefore, the oblique combined score was double-weighted in this study.

### Comparison of malar position and jaws

This study demonstrated that the RM group was considered less attractive than the NM and PM groups, which was consistent with the popular notion that malar hypoplasia leads to poor aesthetic outcomes. Moreover, the PJ group was considered less attractive than the NJ and RJ groups, which was in accordance with the findings of Soh et al. and Chan et al. [27, 28]. Besides, the observers conferred better ratings to the retruded jaws as compared to neutral jaws in the female subject which was in contrast to previous studies by Chan et al. [28] and Soh et al. [27] where Caucasian and Asian dental students (94.6% ethnically Chinese) respectively preferred a normal profile to retruded jaws in the female subject. This difference can be attributed to the changes in certain perceptions of beauty over time, as the present study and the study by Soh et al. although conducted in Asia, but are separated by a 10-year time interval, during which media influence on aesthetic standards [28] might have changed.

### Comparison of the RP, RD and BP groups

Meticulous literature search shows that previous studies focused on the relative positions between the nose, chin, and lips concluding the spatial relationship between them to be crucial for a beautiful face [43–45]. On the other hand, the studies documented on malar are mainly for orthognathic or plastic surgery purposes. To the best of our knowledge, this is the first investigation that documents the aesthetic effects of different malar positions relative to jaws. We believe that the relative alteration in the malar-dentoalveolar region would influence not only the perception of facial profile but also the overall facial aesthetics [11, 46], therefore, the present investigation highlights the effect of relative malar-jaw positions while providing guidance to the professionals not only in the fields of orthodontics and surgery, but also related to facial aesthetics, in making treatment plans that are consistent with the patient expectations, thereby resulting in treatments with aesthetic benefits that can be perceived by all.

According to the results, the observers unanimously considered the RD group (MJC ≤ − 4) to be the most unattractive in the images of both the male and female subjects suggesting a deficient malar relative to the jaws to negatively affect the facial aesthetics. On the other hand, the RP group (MJC ≥ + 4) was generally considered attractive and was even rated to be more attractive than the BP group (MJC = 0) among the images of the male subject. However, when the individual faces were analysed in the RP group, the attractiveness did not seem to increase further as the MJC increased from + 4 to + 8, that is to say, prominence of the malar increased relative to the jaws. Consistent with these observations, the observers identified No. 4 as the most unattractive face in both the sexes, while the overall scores assigned to No. 5 and 7 did not differ significantly. Therefore, based on the findings reported in the present study, it can be inferred that increasingly deficient malar relative to the jaws, is associated with a decreasing level of facial attractiveness. Our findings are in agreement with the literature where several studies have reported that a prominent malar is attractive while deficient malar is considered unattractive [24, 47].

When carefully planned and executed, malar-jaw contouring might result in high patient satisfaction translating into an equally significant positive benefit impacting the patient’s psychosocial environment. In this regard, Leonard and Walker concluded that while planning maxillary advancement, it is indispensable to consider malar prominences for the precise diagnosis of maxillary deficiency [48]. For instance, in the case of combined maxillary-malar deficiency, a LeFort II surgery should be preferred over LeFort I surgery, which would advance not just the maxilla but also the malar prominences, as suggested by Leonard and Walker. Hence, customisation of the type and degree of surgical correction for each facial promontory must be individualised and pre-determined for each patient to enable greater flexibility in achieving optimal results [16].

The relative balance of the facial traits with each other is affected by their strength of the mass and the volume, that are characteristic of each promontory. Patients with maxillary anteroposterior deficiency, generally also have deficient malar with poorly supporting soft tissues in the midfacial region, reason being, the osseous structures are often deficient as a group rather than in isolation [7]. Therefore a more comprehensive relative approach is required before planning facial aesthetic procedures. The ideal amount of malar projection in the sagittal plane (along the Frankfurt horizontal) in relation to cornea has been identified to be approximately 2 mm beyond the anterior surface of the cornea [24, 31]. Likewise, the idea of predicting the amount of malar advancement required in relation to maxilla, has been proposed by Marianetti et.al [23]. However, malar projection in association to the maxillomandibular complex has never been evaluated, hence it was considered worthwhile to perform this study. Based on the present analysis, patients needing surgical correction of the maxillomandibular complex with/without malarplasty, along-with the effect of the amount of surgical correction required, can be envisaged.

### Comparison of images of male and female subjects within groups

The images of the female subject within the RD group were found to be less attractive than the male subject. Notably, the attractiveness rating dropped considerably from BP to RD for the female subject, but not for the male subject, suggestive of the fact that a relatively deficient malar might have a strong negative aesthetic effect on the female faces compared to male faces. The malar group comparisons also supported this conception, as the images of the female subject were considered less aesthetically pleasing than those of the male subject in the RM group.

### Most influential part

Consistent with other studies [49], the present study identified no effect of the observer’s gender on the perception of aesthetics. Further, the analysis of the most influential facial features revealed lips and malar to have the strongest influence on the observer’s decision regarding aesthetics. These results were in agreement with those of Tatarunaite et al. in which the cheeks were strongly associated with the overall attractiveness [35].

The importance of overall balance and harmony among aesthetic units of the face has been emphasized in the studies [50–53]. Correspondingly, the above analysis suggests that the feature of the deficient malar would be more apparent when the jaws are protruded. In contrast, a prominent malar would not standout unless complemented by average jaw protrusions, thereby suggesting that overall facial attractiveness may not be dependent only on a single feature. This idea can be supported by a previous study [35] wherein the malar (cheeks) and the mandible (chin) were found to outweigh all other facial features when determining attractiveness.

From the results of the present study, it can be deduced that profiles with increased convexity resulting from slight malar projection, are considered more aesthetically pleasing, in relation to balanced profiles, suggestive of a direct relationship between relatively prominent malar and aesthetic appearance. In the present study, facial profile analysis enabled the assessment of malar eminence relative to lower facial convexity. The authors advocate the evaluation of malar protrusion in planning the correction for sagittal skeletal discrepancies. Further, this study disclosed that any decrease in the malar projection was associated with the accession of the score assigned to profile aesthetics. Therefore, it can be inferred that women with deficient malar relative to the jaws are particularly less attractive, very often requiring surgical intervention associated with the orthodontic correction to enhance aesthetics.

The findings of this study, suggest that given the aesthetic importance of the malar, orthodontic/orthognathic treatment plans should consider malar projection along with the maxillo-mandibular complex in the anteroposterior dimension. However, the study is limited by the inclusion of only dental students as observers of the facial images. Also, only Asian subjects and observers were involved, therefore other cultural aesthetic standards were not represented. Further studies are required to analyse, whether similar results would be obtained if laymen and professional dentists were included as observers.

## Conclusion

In the present study, retruded malar and protruded jaws were considered aesthetically unattractive facial features. Conversely, neutral malar coupled with retruded jaws was considered most aesthetically appealing in both Chinese male and female subjects. As observed in the present study, an appropriate relationship between malar projection and jaw convexity is decisive with regards to aesthetic facial perception. Therefore, based on the findings of the present study it can be implied that overall facial attractiveness does not rely on a single feature, indeed, relative positions of malar and jaws are equally important. The authors emphasise the importance of examining the malar prominences routinely while performing a facial analysis. In the end, the results of the present study suggest that while designing the orthodontic/orthognathic treatment plans, the relative positions of malar and jaw should be considered for enhanced aesthetic outcomes.

## Data Availability

The datasets used and/or analysed during the current study are available from the corresponding author on reasonable request.
